# From *male PCOS* to male endocrine-metabolic syndrome: a gender-neutral hypothesis

**DOI:** 10.3389/fendo.2026.1903408

**Published:** 2026-08-11

**Authors:** Maurizio Nordio, Daniele Barbaro, Valdemaro Pavacci

**Affiliations:** 1 The Experts’ Group On Inositol In Basic and Clinical Research, and on PMOS (EGOI-PCOS/PMOS), Rome, Italy; 2 Department of Experimental Medicine, Sapienza University, Rome, Italy; 3 U.O. Endocrinology, Livorno Hospital, Livorno, Italy; 4 UniCamillus – Saint Camillus International University of Health Sciences, Rome, Italy

**Keywords:** androgenetic alopecia, cardiometabolic risk, insulin resistance, male endocrine-metabolic syndrome, male PCOS equivalent, PMOS, EMS

## Abstract

The proposed renaming of polycystic ovary syndrome (PCOS) as polyendocrine metabolic ovarian syndrome (PMOS) is an important step toward recognizing the systemic endocrine-metabolic nature of a condition historically misrepresented by ovarian cystic morphology. However, the persistence of the word ovarian remains conceptually limiting and reinforces the perception of a female-exclusive disorder. Published reports have provocatively proposed a male PCOS equivalent, particularly in male relatives of women with PCOS. We posit that those observations may be better conceptualized as male Endocrine-Metabolic Syndrome (male EMS), rather than male PCOS, hoping to offer a more coherent depiction of this clinical condition. In this perspective, *male EMS* is thus proposed as a sex-specific expression of shared endocrine-metabolic alteration characterized by insulin resistance, compensatory hyperinsulinemia, altered sex hormone-binding globulin dynamics, adrenal androgen dysregulation, early-onset androgenetic alopecia, acne or seborrhea, hypertrichosis, cardiometabolic vulnerability, and possible testicular or reproductive abnormalities. Unlike classical metabolic syndrome, male EMS would include endocrine-androgenic and dermatological features and may emerge earlier in life, particularly in individuals with a family history of female PCOS/PMOS/EMS. The present hypothesis remains to be validated prospectively, and male EMS should be considered a conceptual, research-oriented construct, not yet a defined clinical diagnosis. Nevertheless, such framework may help generate testable hypotheses and stimulate early identification of endocrine and cardiometabolic risk in men.

## 1 Introduction

The suggested renaming of polycystic ovary syndrome (PCOS) as polyendocrine metabolic ovarian syndrome (PMOS) represents a pivotal milestone in reproductive and metabolic endocrinology (1). Although still a proposal without global recognition, the key point is that the new terminology acknowledges that the disease is not defined by ovarian cysts and that its clinical burden extends across multiple domains, encompassing endocrine, metabolic, dermatological, psychological, reproductive, and cardiometabolic dysregulations. The change, therefore, shifts the syndrome definition, focusing on systemic pathophysiology.

Nevertheless, PMOS remains a conceptually incomplete effort. While removing the misleading word *polycystic*, the new term retains the word *ovarian*, thus still leading clinicians, researchers, and patients to consider the disorder as intrinsically gender specific and anatomically well defined. Such an interpretation is increasingly difficult to reconcile with literature describing male relatives of women with PCOS who exhibit endocrine, dermatological, and metabolic abnormalities (2–4). Curiously, these observations have led several authors to use the intentionally provocative expression male PCOS or male PCOS equivalent (5, 6).

The expression male PCOS maintains however historical value, challenging the assumption of a sex-specific biology underlying PCOS. Once the condition is reframed as of endocrine-metabolic rather than ovarian origin, the term becomes unnecessary and biologically inaccurate. Men, indeed, express the same endocrine-metabolic alterations through different target organs and clinical signs.

This article hypothesizes the occurrence of a male Endocrine-Metabolic Syndrome (male EMS) as a sex-specific expression of an endocrine-metabolic disorder that, in women, often becomes clinically visible through ovarian alterations, whereas in men it may affect various organs and body districts, including hair follicle, sebaceous gland, adrenal-gonadal axis, testis, adipose tissue, liver, and vasculature. The objective is not to introduce validated diagnostic criteria, but to define a testable clinical and biological framework for future studies.

## 2 Limitations of PMOS as ovarian-centered framework

As mentioned, the introduction of the term PMOS is a significant improvement over PCOS because it acknowledges endocrine and metabolic etiology of the syndrome. However, it stresses out the ovarian involvement, limiting both clinical recognition and research design. In 2023, the group EGOI-PCOS proposed a novel classification that included metabolic deregulation as etiological factor of the syndrome, thus implicitly avoiding its gender specific depiction (7, 8).

Three aspects are particularly relevant:

1. Altered ovarian morphology is not a hallmark of all disease expressions formerly classified as PCOS. In the EGOI-PCOS framework, the hyperandrogenic and insulin-resistant phenotypes formerly classified as Rotterdam phenotypes A, B, and C are reclassified as Endocrine-Metabolic Syndrome (EMS), while the normoandrogenic phenotype D is separated as multi-follicular ovarian disorder (MFOD) (9). This distinction implies that in the systemic hyperandrogenic disorder the ovary is a target organ rather than the origin of the disease.
2. An ovarian-centered name cannot accommodate the male counterpart hypothesis.
3. The use of the term PMOS may delay early risk identification in men who present with early androgenic dermatological signs, low SHBG, elevated DHEAS, visceral adiposity, or insulin resistance, but do not yet meet classical metabolic syndrome criteria.

Therefore, PMOS may be considered a necessary but insufficient nomenclature. While removing the reference to ovarian cysts, it maintains gender specificity; EMS, on the other hand, offers a more biologically flexible terminology because unrestricted to a single gender.

It should be emphasized that current international guidelines and much of the clinical and scientific literature still use PCOS, and the adoption of PMOS has not yet been universally agreed upon.

In this manuscript, the terms PCOS, PMOS, and EMS are therefore used to acknowledge both the established guideline terminology and the emerging nomenclatures.

## 3 From male PCOS to the hypothesis of a male EMS research framework

The term *male PCOS equivalent* has been used in the literature to describe endocrine and metabolic abnormalities in men related to women with PCOS. Cannarella and colleagues discussed whether a male PCOS equivalent exists and proposed that the genetic background of PCOS could define a male counterpart (5), while Di Guardo and colleagues reviewed hormonal, metabolic, and clinical aspects of this potential male equivalent (6). More recent family-based reviews and meta-analyses have further supported the plausibility of endocrine-metabolic clustering in male first-degree relatives of women with PCOS, while also emphasizing the absence of validated diagnostic criteria (2, 10).

The 2025 review on EMS and metabolic syndrome makes this conceptual shift clearer, stating that one critical difference between Rotterdam and EGOI-PCOS criteria is that EMS does not exclude male patients (11). The same review describes men with EMS-like features as having metabolic disturbances similar to those of women with EMS, including insulin resistance, dyslipidemia, cardiovascular predisposition, variations in androgen and SHBG levels, and clinical signs such as early-onset balding, acne, and hypertrichosis.

The proposed male EMS research framework has three advantages: a) it removes the contradiction inherent in male PCOS; b) it preserves the endocrine-metabolic involvement of the disorder; c) it allows male and female phenotypes to be studied as sex-specific expressions of shared endocrine-metabolic alterations rather than as unrelated diseases.

## 4 Proposed conceptual framework for male EMS

We hypothesize that male Endocrine-Metabolic Syndrome should be interpreted as a sex-specific expression of an inherited or acquired endocrine-metabolic alteration characterized by the following features: insulin resistance; compensatory hyperinsulinemia; altered androgen/SHBG dynamics; adrenal androgen dysregulation; early-onset androgenetic alopecia, acne or seborrhea; hypertrichosis; cardiometabolic vulnerability; in some cases, altered testicular steroidogenesis, or reproductive parameters.

This proposed definition would avoid reducing the condition to classical metabolic syndrome. In other words, male EMS would not be simply limited to obesity, dyslipidemia, or impaired glucose tolerance, it would consist of a broader pattern in which metabolic dysfunction interacts with androgenic, dermatological, adrenal, and possibly reproductive manifestations.

We suggest that a general clinical rule could be formulated as follows: male EMS is to be reasonably suspected when a male patient presents metabolic dysfunction plus androgenic or dermatological signs, particularly in the presence of a family history of female EMS/PMOS/PCOS.

It is important reminding that this should be considered a hypothesis-generating definition, designed for research stratification and early clinical recognition, not as validated set of diagnostic criteria.

## 5 Pathophysiological model

The proposed male EMS hypothesis would be based on known interacting mechanisms:

• **Insulin sensitivity**

In female EMS/PMOS/PCOS, hyperinsulinemia may stimulate ovarian androgen production because the ovary remains sensitive to insulin even when other tissues are insulin resistant (12). At the same time, hyperinsulinemia reduces production of SHBG, thus increasing androgen bioavailability and contributing to hyperandrogenism and follicular dysfunction (13–16). Conversely, the same metabolic environment may generate a different response of the hypothalamic-pituitary-gonadal axis in men. Indeed, deregulation of insulin sensitivity, adiposity, inflammation, and gonadal feedback may result into altered LH/testosterone dynamics that change Leydig cell function, adrenal androgen production, SHBG synthesis, and tissue-level androgen sensitivity (17).

Therefore, male EMS would not be expected to mirror female EMS, and other markers and signs might be more informative than total testosterone alone.

• **Local androgen sensitivity**

Androgenetic alopecia, acne, seborrhea, and hypertrichosis may depend not only on circulating androgen concentrations, but also on local androgen receptor sensitivity, 5-alpha-reductase activity, sebaceous gland biology, and hair-follicle responsiveness (18). This could explain why androgen-sensitive dermatological signs may coexist with normal or even reduced circulating testosterone in metabolically unhealthy men.

• **Adipose tissue**

In female EMS/PMOS/PCOS, adipose tissue dysfunction has been related to metabolic disorders associated to inflammation and oxidative stress (15, 19). Whether analogous adipose-tissue mechanisms contribute to a potential male EMS phenotype remains unknown, but they are biologically plausible pathways to test given their role in the female counterpart (20).

• **Genetic and epigenetic susceptibility**

A polygenic risk score for PCOS has been associated in men with cardiometabolic and endocrinologic alterations (21). These findings support the possibility that some genetic risk related to EMS/PMOS/PCOS can be independent of ovarian function, although effect sizes are modest and mechanisms remain incompletely understood. The involvement of genetic factors in the etiology of EMS is further corroborated by evidence that altered epigenetic regulation contributes to intergenerational susceptibility (22).

• **Gut microbiome**

Sex steroids and metabolic status can influence microbial composition, and gut dysbiosis has been proposed as a contributor to PCOS-related endocrine and metabolic alterations (23). Whether the entity of such alteration is relevant in men is still unknown, but variations in the composition of gut microbiome are expected to worsen endocrine-metabolic dysfunction.

The gender divergence that arises is central to the proposed model. In women, the ovary may express endocrine-metabolic dysfunction through oligo-anovulation, ovarian androgen excess, infertility, and multi-follicular morphology; in men, the same alteration may affect hair follicle sensitivity, sebaceous activity, adrenal androgen patterns, SHBG levels, cardiometabolic risk, and, in some cases, testicular or semen physiology (24).

## 6 Proposed clinical phenotypes of male EMS

**Table 1** summarizes the main features of male EMS for each relevant proposed domain.

**Table 1:** Proposed clinical profile of male endocrine-metabolic syndrome (EMS).

Domain	Possible male EMS features (to be investigated)	Clinical interpretation
Dermatological	Early-onset androgenetic alopecia, acne, seborrhea, hypertrichosis, acanthosis nigricans	Visible androgenic or insulin-resistant phenotype
Metabolic	Insulin resistance, hyperinsulinemia, visceral adiposity, dyslipidemia, impaired glucose tolerance, MASLD, early hypertension	Metabolic core of the syndrome - differential diagnosis against Metabolic Syndrome is relevant
Hormonal	Low SHBG, elevated DHEAS, low FAI, normal or low testosterone in insulin-resistant men, altered LH/FSH dynamics	Male-specific androgen/SHBG pattern
Cardiovascular	Early hypertension, endothelial risk, family history of cardiovascular disease	Early cardiometabolic vulnerability
Liver/adipose tissue	Metabolic dysfunction-associated steatotic liver disease, central adiposity, adipokine and inflammatory abnormalities	Metabolic progression and risk amplification
Reproductive	Altered semen parameters, functional hypogonadism, Leydig cell dysfunction, altered LH/FSH dynamics	Exploratory research endpoint requiring validation
Familial/genetic	Female first-degree relatives with EMS/PMOS/PCOS, PCOS polygenic score – if available	Supports shared endocrine-metabolic susceptibility
Exclusions	Anabolic steroid use, androgen exposure, adrenal tumors, congenital adrenal hyperplasia, Cushing syndrome, primary hypogonadism, pituitary-gonadal disease, uncontrolled thyroid disease	Avoids misclassification

### 6.1 Dermatological-androgenic signs

Dermatological signs are suggested to be among the most visible candidate features of the proposed male EMS framework. Early-onset androgenetic alopecia, especially before 35 years of age, may represent a clinically visible marker of endocrine or metabolic susceptibility. Acne, seborrhea, hypertrichosis, and acanthosis nigricans may also be relevant signs when insulin resistance or familial EMS/PMOS/PCOS is present.

It is important to point out that these signs can characterize otherwise healthy men. Isolated androgenetic alopecia, acne, seborrhea, or hypertrichosis cannot define the proposed phenotype. Their relevance should therefore be considered only in combination with metabolic, endocrine, and familial variables, and not used as stand-alone diagnostic markers.

### 6.2 Metabolic signs

The metabolic components of male EMS may include the following: fasting or post-load hyperinsulinemia; elevated HOMA-IR; impaired glucose tolerance; visceral adiposity; dyslipidemia; metabolic dysfunction-associated steatotic liver disease; early hypertension; and increased lifetime cardiovascular risk. These features overlap with metabolic syndrome but should be interpreted together with endocrine-androgenic signs within the proposed male EMS framework (11).

### 6.3 Hormonal signs

Potential hormonal markers include: low SHBG; low FAI; elevated DHEAS; increased androstenedione in selected cases; normal, low, or high testosterone depending on age; adiposity; insulin resistance; and reduced testicular response. In severe metabolic dysfunction, functional hypogonadism may coexist with androgen-sensitive dermatological signs. This apparent paradox is compatible with tissue-specific androgen sensitivity and differential adrenal versus testicular contributions.

### 6.4 Reproductive and testicular features

The reproductive aspect of the proposed male EMS framework remains insufficiently defined. Possible research endpoints include semen concentration, motility, morphology, total motile sperm count, sperm DNA fragmentation, Leydig cell function, LH/FSH dynamics, and idiopathic infertility associated with insulin resistance or visceral adiposity.

## 7 Proposed male EMS framework versus classical metabolic syndrome

As mentioned, male EMS must be distinguished from classical metabolic syndrome (**Table 2**).

**Table 2:** Male endocrine-metabolic syndrome (EMS) versus classical metabolic syndrome.

Feature	Proposed male EMS framework	Classical metabolic syndrome
Primary nature	Endocrine-metabolic disorder	Cardiometabolic risk cluster
Core mechanism	Insulin resistance plus androgen/SHBG and adrenal-gonadal dysregulation	Central obesity plus glucose, lipid, and blood pressure abnormalities
Androgen involvement	Central or strongly suggestive	Not diagnostic
Dermatological signs	Early-onset androgenetic alopecia, acne, seborrhea, hypertrichosis	Not defining
SHBG	Often reduced or altered	May be reduced, but not part of diagnostic criteria
DHEAS	May be elevated or dysregulated	Not part of diagnostic criteria
Reproductive axis	Possible semen or testicular steroidogenic abnormalities	Not defining
Family history	Female relatives with EMS/PMOS/PCOS may be relevant	Family history of diabetes or cardiovascular disease more relevant
Age of manifestation	May begin in adolescence or young adulthood	More common later, but can occur earlier
Clinical status	Not validated; research framework only	Validated risk construct used clinically

Metabolic syndrome consists of cardiometabolic risk cluster defined by central obesity, dyslipidemia, hypertension, and impaired glucose regulation. The proposed male EMS framework, on the other hand, is defined by an endocrine-metabolic disorder in which insulin resistance interacts with androgenic, dermatological, adrenal, and - possibly - reproductive features.

The distinction might assume clinical relevance because male EMS would be detected before full metabolic syndrome develops, thus allowing earlier intervention and endocrine-metabolic risk reduction.

According to the EGOI-PCOS position that EMS and metabolic syndrome are distinct but interrelated pathologies (11), male EMS is thought to precede, overlap with, or evolve into metabolic syndrome.

## 8 Proposed male EMS versus female EMS/PMOS/PCOS

A gender-neutral framework would not imply that the disorder is identical in men and women, it rather proposes that a shared endocrine-metabolic alteration may be expressed through different sex-specific target organs. The ovary is one target organ in women – it should not define the disease in men.

The difference between male and female EMS (**Table 3**) is essential when it comes to interpreting androgen levels. In women, hyperinsulinemia may promote ovarian androgen production and reduce SHBG, leading to biochemical and clinical hyperandrogenism. In men, insulin resistance may coexist with normal or low testosterone because of obesity-related hypogonadism or insulin-mediated effects on Leydig cells, while altered DHEAS, SHBG, and local androgen sensitivity may still generate dermatological signs.

**Table 3:** Female EMS/PMOS/PCOS versus proposed male endocrine-metabolic syndrome (EMS) model.

Domain	Female EMS/PMOS/PCOS	Proposed male EMS framework
Nomenclature problem	PMOS improves on PCOS but remains ovarian-centered. PCOS is still largely used, while PMOS needs universal validation	PMOS/PCOS exclude male syndrome
Gonadal target organ	Ovary	Testis, adrenal-gonadal axis, skin/hair follicle
Reproductive signs	Oligo-anovulation, infertility	Possible altered semen quality or testosterone dynamics
Androgenic signs	Hirsutism, acne, alopecia	Early-onset androgenetic alopecia, acne, seborrhea, hypertrichosis
Metabolic signs	Insulin resistance, obesity, dyslipidemia	Insulin resistance, visceral adiposity, dyslipidemia
SHBG	Often reduced	Often reduced or altered
DHEAS	May be elevated	May be elevated
Testosterone	Often elevated	Not necessarily elevated; may be normal or low
Cardiometabolic risk	Increased	Increased

## 9 Candidate research domains for the proposed male EMS framework

The following domains are not diagnostic criteria. They are candidate research domains intended to support hypothesis generation, study design, and prospective validation. Mixed phenotypes are expected, and no domain should be interpreted as sufficient for clinical diagnosis.

### 9.1 Domain A: metabolic component

- HOMA-IR above the locally validated insulin resistance threshold, for example >2.5 in studies using this cutoff;
- fasting hyperinsulinemia or abnormal insulin response during an oral glucose tolerance test;
- impaired fasting glucose, impaired glucose tolerance, or elevated HbA1c;
- visceral adiposity, dyslipidemia, MASLD, or early hypertension.

### 9.2 Domain B: androgenic, dermatological, or endocrine component

- early-onset androgenetic alopecia, especially before 35 years of age – only when combined with metabolic, endocrine, or familial features;
- persistent acne, seborrhea, hypertrichosis, or acanthosis nigricans when not isolated;
- low SHBG, elevated DHEAS, low FAI, or adrenal androgen dysregulation;
- functional hypogonadal pattern in the presence of insulin resistance or visceral adiposity.

### 9.3 Domain C: familial or genetic clues

- mother, sister, daughter, or other first-degree female relative with EMS/PMOS/PCOS;
- family history of female hyperandrogenism, early type 2 diabetes, or premature androgenetic alopecia associated with dysmetabolism;
- polygenic risk score for PCOS when available.

### 9.4 Domain D: exclusion and differential diagnosis

- anabolic steroid use or exogenous androgen exposure;
- primary hypogonadism or known pituitary-gonadal disease;
- classical or non-classical congenital adrenal hyperplasia;
- androgen-secreting tumors or Cushing syndrome;
- uncontrolled thyroid disease;
- medications significantly altering androgen or SHBG levels;
- isolated dermatological signs of hyperandrogenism without metabolic or endocrine abnormalities.

Proposed general rule: for research purpose, male EMS is to be suspected when a male patient presents a metabolic component plus an androgenic, dermatological, or endocrine component, especially when a first-degree female relative has EMS/PMOS/PCOS.

## 10 Clinical and research implications

If validated, the proposed male EMS framework might have several implications: first, it may support earlier cardiometabolic prevention in young men who do not yet meet metabolic syndrome criteria; second, it may help identify familial endocrine-metabolic clusters; third, it may improve interpretation of male infertility associated with metabolic dysfunction; fourth, it may support gender-neutral genetic, metabolomic, microbiome, and endocrine research.

## 11 Research agenda

The male EMS hypothesis can be tested through cross-sectional, longitudinal, genetic, and interventional studies. Priority populations include brothers, fathers, and sons of women with EMS/PMOS/PCOS; men with early-onset androgenetic alopecia; men with acne or seborrhea plus insulin resistance; men with idiopathic infertility and dysmetabolism; and men with low SHBG and visceral adiposity.

Suggested biomarkers include fasting insulin, insulin response during oral glucose tolerance testing, HOMA-IR, HbA1c, lipid profile, liver enzymes, liver ultrasound, SHBG, total testosterone, calculated FAI, free testosterone, DHEAS, androstenedione, LH, FSH, semen parameters, inflammatory markers, adipokines, microbiome profile and polygenic risk scores when available.

Important study designs include: prospective studies of male first-degree relatives of women with EMS/PMOS/PCOS; longitudinal studies of early-onset androgenetic alopecia and later metabolic disease; case-control studies comparing male EMS candidates with classical metabolic syndrome; reproductive studies in men with idiopathic infertility and insulin resistance; and genetic studies testing shared risk loci across female EMS and male EMS.

## 12 Discussion

The male EMS hypothesis challenges the ovarian-centered dimension of female EMS/PMOS/PCOS. The proposed change from PCOS to PMOS, should it be largely adopted, does not fully correct the anatomical restriction; if men can express related endocrine-metabolic alterations through male-specific target organs, then the disorder should not be conceptually defined by the ovaries.

The term male PCOS was useful as a provocation because it made this contradiction visible. However, it is biologically inaccurate and should be replaced, at least in research contexts, by a more gender-neutral and mechanism-oriented term. *Male EMS* is proposed for this purpose, while recognizing that it remains provisional and potentially confusing unless carefully distinguished from classical metabolic syndrome.

One limitation of the hypothesis, indeed, is that endocrine and metabolic alterations are key distinctive characteristics of Metabolic Syndrome, and current evidence are insufficient to demonstrate that male EMS is a separate disease entity. It may ultimately represent a subgroup of metabolic syndrome with androgenic or adrenal manifestations, present along with other risk factors. Distinctiveness would be supported only with future studies demonstrating reproducible clustering, family or genetic enrichment related to EMS/PMOS/PCOS, incremental predictive value beyond classical metabolic syndrome, and prospective association with cardiometabolic or reproductive outcomes.

Moreover, overdiagnosis may represent a central risk, as isolated signs would not be sufficient for male EMS definition, thus classification into clinical phenotypes should be interpreted with caution.

At this stage, formal validation of the male EMS hypothesis would require prospective cohorts, standardized phenotyping, comparison with classical metabolic syndrome, genetic and epigenetic profiling, mechanistic studies, and independent replication. It would also require consensus from international endocrine, metabolic, reproductive, dermatological, and andrological societies before any formal nomenclature or diagnostic criteria could be proposed.

The strength of the hypothesis, on the other hand, lies in its testability, directly evaluating predictions of endocrine-metabolic disorders in man with first-degree female relatives with EMS/PMOS/PCOS.

## 13 Conclusion

PMOS is a relevant step forward with respect to PCOS, but its implementation remains in transition and current guidelines still largely use PCOS terminology. Moreover, PMOS remains ovarian-centered and is therefore incomplete as a gender-neutral conceptual framework.

The male counterpart hypothesis suggests that the endocrine-metabolic susceptibility linked to PCOS/PMOS is systemic and expressed through sex-specific target organs in males and females. The term *male PCOS* is to be considered historically useful but conceptually obsolete, with *male Endocrine-Metabolic Syndrome* (male EMS) as a more coherent provisional research term, provided it is not confused with classical metabolic syndrome.

This proposed male EMS framework is hypothesized to include metabolic and hormonal dysfunctions, with candidate manifestations such as early-onset androgenetic alopecia, acne, seborrhea, hypertrichosis, cardiometabolic vulnerability, and possible testicular or reproductive abnormalities. However, these features are not diagnostic and require prospective validation.

Male EMS should therefore be regarded as a hypothesis-generating framework, and not as validated clinical syndrome. Its value will depend on whether future studies demonstrate reproducible clustering, familial and genetic enrichment, mechanistic plausibility, and predictive utility beyond classical metabolic syndrome.

## Data Availability

The original contributions presented in the study are included in the article/supplementary material, further inquiries can be directed to the corresponding author/s.
